# Relation between reperfusion and hemorrhagic transformation in acute ischemic stroke

**DOI:** 10.1007/s00234-015-1577-6

**Published:** 2015-09-04

**Authors:** Alexander D. Horsch, Jan Willem Dankbaar, Yolanda van der Graaf, Joris M. Niesten, Tom van Seeters, Irene C. van der Schaaf, L. Jaap Kappelle, Birgitta K. Velthuis, On behalf of the DUST investigators

**Affiliations:** Department of Radiology, University Medical Center Utrecht, Heidelberglaan 100, HP E01.132, 3584 CX Utrecht, The Netherlands; Department of Radiology, Rijnstate Hospital, Arnhem, The Netherlands; Julius Center for Health Sciences and Primary Care, Utrecht, The Netherlands; Department of Neurology, Utrecht Stroke Center, University Medical Center, Utrecht, The Netherlands

**Keywords:** Ischemic stroke, CT perfusion, rtPA, Reperfusion, Hemorrhagic transformation

## Abstract

**Introduction:**

Intravenous recombinant tissue plasminogen activator (IV-rtPA) is given in acute ischemic stroke patients to achieve reperfusion. Hemorrhagic transformation (HT) is a serious complication of IV-rtPA treatment and related to blood–brain barrier (BBB) injury. It is unclear whether HT occurs secondary to reperfusion in combination with ischemic BBB injury or is caused by the negative effect of IV-rtPA on BBB integrity. The aim of this study was to establish the association between reperfusion and the occurrence of HT.

**Methods:**

From the DUST study, patients were selected with admission and follow-up non-contrast CT (NCCT) and CT perfusion (CTP) imaging, and a perfusion deficit in the middle cerebral artery territory on admission. Reperfusion was categorized qualitatively as reperfusion or no-reperfusion by visual comparison of admission and follow-up CTP. Occurrence of HT was assessed on follow-up NCCT. The association between reperfusion and occurrence of HT on follow-up was estimated by calculating odds ratios (ORs) and 95 % confidence intervals (CIs) with additional stratification for IV-rtPA treatment.

**Results:**

Inclusion criteria were met in 299 patients. There was no significant association between reperfusion and HT (OR 1.2 95%CI 0.5–3.1). In patients treated with IV-rtPA (*n* = 203), the OR was 1.3 (95%CI 0.4–4.0), and in patients not treated with IV-rtPA (*n* = 96), the OR was 0.8 (95%CI 0.1–4.5). HT occurred in 14 % of the IV-rtPA patients and in 7 % of patients without IV-rtPA (95%CI of difference −1 to 14 %).

**Conclusion:**

Our results suggest that the increased risk of HT after acute ischemic stroke treatment is not dependent on the reperfusion status.

## Introduction

Timely restoration of the downstream capillary blood flow (reperfusion) by recanalization of the occluded vessel in acute ischemic stroke patients is associated with favorable clinical outcome [1, 2]. However, reperfusion has also been associated with the occurrence of hemorrhagic transformation (HT) through a mechanism called reperfusion injury [1, 3–5]. HT incorporates all types of post-ischemic hemorrhages, ranging from the smaller hemorrhagic infarction (HI) type 1 or 2, to the larger parenchymal hemorrhage (PH) type 1 or 2. Especially, the PH types may increase the risk of worse clinical outcome [6–9]. To induce reperfusion and thereby improve clinical outcome, intravenous recombinant tissue plasminogen activator (IV-rtPA) can be given within 4.5 h after symptom onset [10]. However, IV-rtPA also increases the risk of HT by its thrombolytic action as well as by causing direct damage to the blood–brain barrier (BBB) [10–13]. It is unclear whether HT after ischemic stroke is caused mainly by reperfusion of an ischemic area with damage to the BBB or by the detrimental effects of IV-rtPA on the BBB [3, 9]. To further explore this topic, first the relation between reperfusion and the occurrence of HT needs to be investigated.

The purpose of this study was to investigate the association between reperfusion and the occurrence of HT, both in patients treated with and without IV-rtPA.

## Materials and methods

### Study design

The Dutch acute stroke study (DUST) is a large prospective multicenter cohort study that aims to assess the value of CT perfusion (CTP) and CT angiography (CTA) in addition to patient characteristics and non-contrast CT (NCCT) for prediction of outcome in patients with acute ischemic stroke (ClinicalTrials.gov NCT00880113). Patients were included in 14 hospitals between May 2009 and August 2013 [14].

Inclusion criteria for the DUST were as follows: age >18 years, suspected ischemic stroke of less than 9 h in duration, and National Institutes of Health Stroke Scale (NIHSS) ≥2, or 1 if an indication for IV-rtPA was present. Exclusion criteria were known renal failure, contraindications for iodinated contrast material, and hemorrhage or another diagnosis seen on NCCT to explain the stroke symptoms. This study was approved by the central medical ethics committee in the University Medical Center (UMC) Utrecht and the local institutional ethical review boards of the participating hospitals. Patients or family gave signed informed consent unless a patient died before consent could be obtained, in which case the need for consent was waived by the medical ethics committee [14].

### Patient selection

**Fig. 1:**
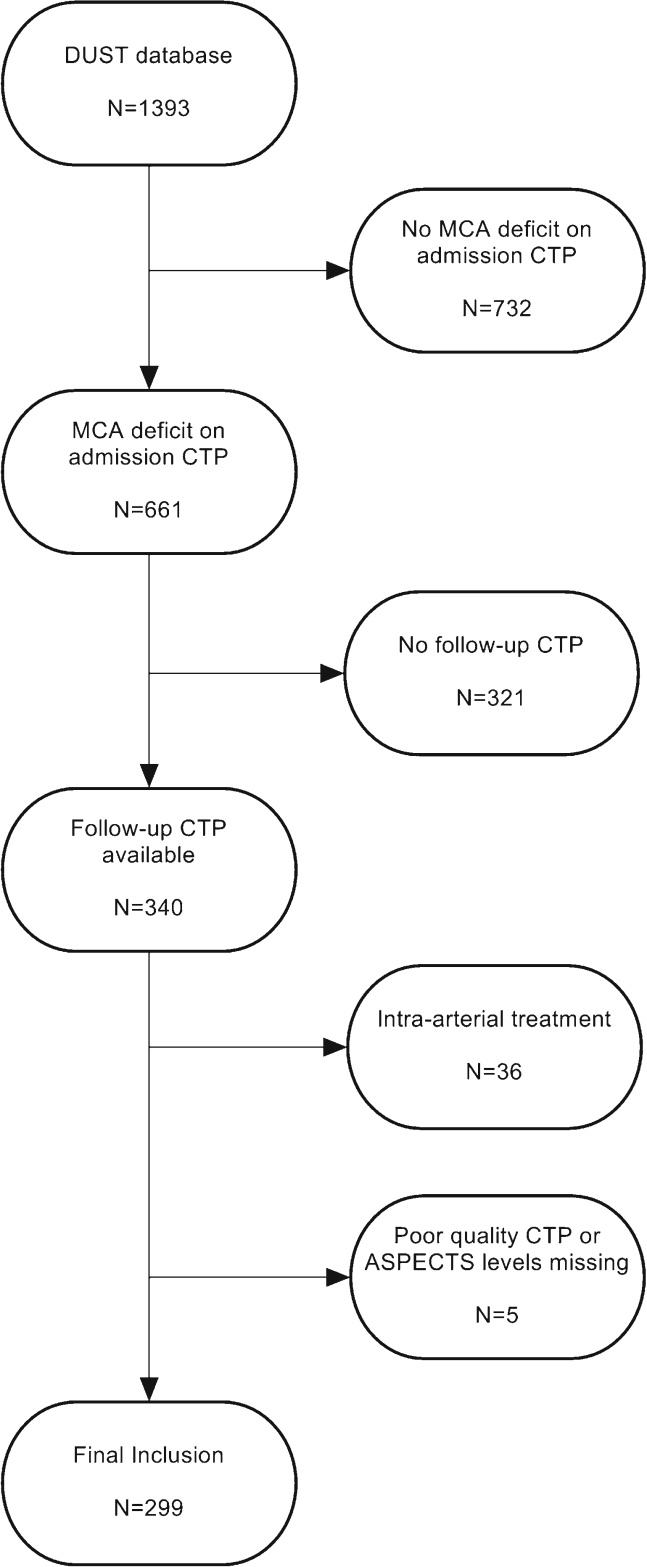
Flow chart patient selection

From the prospectively collected DUST database, patients were retrospectively selected with (1) admission and follow-up NCCT and CT perfusion and (2) a perfusion deficit in the middle cerebral artery territory on admission CTP. Exclusion criteria were as follows: (1) intra-arterial treatment, (2) poor quality CTP, or (3) absence of one of the Alberta Stroke Program Early CT Score (ASPECTS) levels on CTP. The inclusion process is clarified in the flow chart (Fig. 1). Collected clinical data were age, sex, history of stroke or hypertension, NIHSS on admission, IV-rtPA treatment, and time from symptom onset to admission CT scan series.

### Imaging protocol and post-processing

In all DUST patients, NCCT, CTP, and CTA were performed on admission. Non-obligatory follow-up NCCT was planned after 3 days (±2 days) and also performed in case of clinical deterioration. Additional follow-up CTA and CTP were performed if possible. For the current study, patients were included only if follow-up imaging was performed with NCCT and CTP. All imaging studies were performed on multidetector CT scanners ranging from 40 to 320 detectors. The CT protocol has been described previously [14].

In short, the CTP involved successive gantry rotations in cine mode during intravenous administration of iodinated contrast material (40 ml non-ionic contrast) followed by 40 ml of saline, both with a flow of 6 ml/s. The CTP covered at least the level of the basal ganglia to the lateral ventricles to be able to assess ASPECTS levels 1 and 2, and compare between scanners with different number of detectors [15].

From the acquired CTP data, color maps were created for cerebral blood volume (CBV), cerebral blood flow (CBF), mean transit time (MTT), and time to peak (TTP) utilizing commercially available CTP software (Extended Brilliance workstation 4.5, Philips Healthcare). This software uses a deconvolution-based method which determines the MTT by the difference in first moment of tissue and arterial time attenuation curves [16, 17]. To calculate the CBF from the MTT, the software applies the central volume principle which is the most accurate for low injection rates of iodinated contrast material [18]. The internal carotid artery (if available in the scan range) or anterior cerebral artery was chosen as arterial input function [19]. The superior sagittal sinus was used as venous output function.

All data and imaging processing was done centrally in the UMC Utrecht. Scans were evaluated by one of three observers with more than 5 years of stroke imaging experience (B.K.V., I.C.vd.S, J.W.D.). The side of symptoms was provided, but observers were blinded to other clinical and imaging data. Consensus was reached for ambiguous findings by an extra review by two of the three radiologists, also blinded to other clinical and imaging data. The number of consensus agreement cases was not collected.

### Reperfusion

Reperfusion status was analyzed by visual comparison of the size of the perfusion abnormality on admission and follow-up CTP maps for CBV, CBF, MTT, and TTP. Reperfusion was classified qualitatively in a reperfusion and a no-reperfusion group. No-reperfusion was defined as the absence of any visually apparent change in the size of the perfusion deficit on follow-up CTP compared to the admission CTP. Partial reperfusion and hyperperfusion were included in the reperfusion group and an enlarged or new perfusion deficit in the no-reperfusion group.

### Hemorrhagic transformation

The follow-up NCCT was evaluated for the presence of hemorrhage. Hemorrhages were classified according to the European Cooperative Acute Stroke Study (ECASS)-1 criteria: HI-1 (small petechiae along the margins of infarct), HI-2 (confluent petechiae within infarcted area but no space-occupying effect), PH-1 (blood clots in 30 % or less of the infarcted area with some slight space-occupying effect), and PH-2 (blood clots of more than 30 % of infarcted area with substantial space-occupying effect) [20, 7].

### Statistical analysis

Patient characteristics were presented as number and percentages, mean and standard deviation (SD), or median and inter-quartile range (IQ). Differences between patients with and without IV-rtPA treatment were tested with the χ^2^ test to compare categorical variables and the Mann–Whitney *U* test for continuous variables.

The primary outcome was occurrence of any HT (all ECASS categories together). We estimated the association between reperfusion and the occurrence of HT by calculating odds ratios with 95 % confidence intervals (95%CIs) in the total group of patients and in the sub-groups with and without IV-rtPA treatment.

## Results

From the DUST database of 1393 patients with complete admission data, 299 patients met inclusion criteria for this study. A total of 36 HTs (12 %) occurred in this selected patient group (ECASS HI-1, 13; HI-2, 15; PH-1, 7; and PH-2, 1). The overall percentage was comparable to the percentage of HT in the whole DUST database (11 %). The main reasons for exclusion was the absence of an ischemic deficit on admission CTP (*n* = 732) or because no follow-up CTP imaging was done (*n* = 321). No significant difference was found in 3-month mRS between included and all excluded patients.

Clinical and imaging characteristics are summarized in Table 1. Treatment with IV-rtPA was given in 203 of the 299 patients (68 %). Patients treated with IV-rtPA less often had a prior history of stroke or hypertension, had a significantly higher median NIHSS on admission (9 vs 6), and had a shorter median time to scan than patients who did not receive IV-rtPA (84 vs 218 min).

**Table 1:** Baseline clinical and imaging characteristics

	All patients *N* = 299	rtPA *N* = 203	No rtPA *N* = 96	*P* value
Clinical parameters				
Age, median (IQ)	68 (58–77)	68 (56–75)	70 (61–78)	0.10
Female sex, *n* (% )	116 (39)	79 (39)	37 (39)	0.95
Prior stroke, *n* (% )	68 (23)	35 (17)	33 (34)	0.001*
Hypertension, *n* (% )	154 (52)	95 (47)	59 (62)	0.02*
NIHSS, median (IQ)	8 (5–14)	9 (6–15)	6 (3–12)	0.002*
Imaging parameters				
Time to admission scan, minutes, median (IQ)	103 (65–163)	84 (60–133)	218 (133–315)	0.006*
NCCT				
HT, *n* (%)	36 (12)	29 (14)	7 (7)	0.08
CTP				
No reperfusion, *n* (%)	58 (19)	34 (17)	24 (25)	0.09

χ^2^ test was used to compare categorical variables and Mann–Whitney *U* test for continuous variables

*CTP* CT perfusion, *HT* hemorrhagic transformation, *IQ* interquartile range, *NCCT* non-contrast CT, *NIHSS* National Institutes of Health Stroke Scale, *rtPA* recombinant tissue plasminogen activator

*P < 0.05

Reperfusion (median assessed at day 3) was visible in 241 patients (81 %). There was no significant association between HT and reperfusion status in all patients (odds ratio (OR) 1.2, 95%CI 0.5–3.1). Of the 36 (12 %) patients with HT, 29 received IV-rtPA treatment and 7 did not. HT occurred twice as often in patients treated with IV-rtPA compared to patients not treated with IV-rtPA (14 vs 7 % with a 95%CI −1 to 14 % for the difference). Both in patients treated with IV-rtPA and in patients not treated with IV-rtPA, there was no significant association between reperfusion and HT (OR 1.3 (95%CI 0.4–4.0) and OR 0.8 (95%CI 0.1–4.5), respectively).

The results of a sub-analysis show that of the 28 patients with HI-type hemorrhages, 25 (89 %) showed reperfusion, while 3 (11 %) did not. Of the eight patients with PH type hemorrhage, five (63 %) showed reperfusion while three (37 %) did not.

## Discussion

The main finding in this study is that the overall occurrence of hemorrhagic transformation 3 days after onset of acute ischemic stroke does not seem to be associated with reperfusion.

Our findings are in contradiction to Fiehler et al. 2005 who suggested, in a retrospective MRI study in which HT occurred in 19 of the 51 patients, that HT in patients treated with IV-rtPA might be caused by a higher incidence of local reperfusion in the HT area [3]. However, in this study, no significant difference in the occurrence of reperfusion between patients with and without HT could be shown if the entire admission perfusion abnormality area was considered. Moreover, the definition of reperfusion used in that study was only based on changes in TTP delay measured with MRI instead of CT, which could lead to differences in measuring the infarct core and penumbra and hence to different results [3]. Another retrospective MRI study, with HT occurring in 22 of the 144 patients, stated that reperfusion was the most significant independent predictor of early BBB disruption and that this BBB disruption was an independent predictor of HT [21]. However, they did not show a direct relation between reperfusion and HT. BBB disruption was defined as post–gadolinium CSF enhancement, a technique not frequently used in clinical practice. Moreover, only 25 % of their patients received IV-rtPA, and no significant association between IV-rtPA and HT was shown [21].

To our knowledge, our study is the largest prospectively collected dataset to evaluate the association between reperfusion and HT. The overall percentage of HT patients in our study was within the range of previously published data on CT follow-up literature [12, 22]. There was a clear difference in the occurrence of HT between patients treated with IV-rtPA and patients not treated with IV-rtPA, despite the absence of an association with reperfusion. This might be an indication that the delivery of rtPA to the ischemic area and not the reperfusion itself results in HT. However, other factors like stroke severity or time to treatment may also play a role.

Treatment with IV-rtPA was given in 68 % of our patients. This percentage is much higher compared to that of other studies and possibly reflects the increased stroke awareness in The Netherlands and subsequent shorter time to admission. A selection bias in the DUST study with preference given to inclusion of patients eligible for IV-rtPA treatment could also be the reason for this higher percentage of treated patients.

The results of the sub-analysis of the HI-type and PH-type hemorrhages suggest that there is a trend toward a higher perfusion rate in patients developing HI-type hemorrhages, while PH-type hemorrhages seem less related to reperfusion. This is in accordance with the findings of ECASS-2 which showed better outcomes with HI-type hemorrhage and worse outcomes with PH-type hemorrhages, possibly related to reperfusion status [23]. Unfortunately, the numbers in the sub-analysis are too low to perform meaningful statistical analysis on these sub-groups. As we already derived our population from the whole DUST database, we were unable to test this in a larger cohort of patients.

Although we did not show a relation between reperfusion and the occurrence of hemorrhagic transformation, it is possible that this is due to the fact that the exact location of the hemorrhage is difficult to ascertain and that the reperfusion in a very focal area could be of importance. It could be argued that with the use of higher-resolution thin-sliced CTP and added filtering and noise reduction, this relationship could be better determined in future studies [24].

This study has some limitations. First, although the overall number of hemorrhages was comparable to previously published data, the number of symptomatic hemorrhages (defined as PH-2) in this series is low compared to that in some other studies [25, 22]. Most symptomatic hemorrhages (*n* = 23) are not included because the inclusion criteria for this study required a CTP at follow-up. Patients with symptomatic HT may have been too agitated or hemodynamically unstable to lie still long enough to perform this follow-up CTP, and many of the PH-2 outcomes from the DUST database could not be included in this analysis because follow-up CTP was missing. Another explanation for the low number of PH-2’s may be the relatively short time to treatment in our study. It is known that longer time to treatment is related to occurrence of HT [25]. We included smaller hemorrhages (HI-1 and HI-2) in our analysis since they are also related to poor outcome in larger studies [26–28]. Nevertheless, due to the low number of PH-2, our results must be interpreted with caution and are mainly applicable for populations with smaller hemorrhages.

Second, the timing of most follow-up scans was around 3 days. Reperfusion and recanalization are known to continue up to several weeks but the “time is brain” concept states that reperfusion is only relevant if it occurs within several hours after onset of ischemia [29]. It could be argued that reperfusion measurement at an earlier time point would be more appropriate. But, for practical purposes and to minimize discomfort for participating patients, we had to compromise between timing of HT detection and early reperfusion assessment. Moreover, because patients did receive an additional NCCT in case of clinical deterioration after the scheduled follow-up scans were made, it is unlikely that significant hemorrhages were missed.

Third, we excluded patients with intra-arterial treatment as most of these patients were treated with a combination of mechanical thrombectomy and intra-arterial thrombolysis, which probably has an added but unknown effect on the integrity of the BBB.

Fourth, reperfusion status was not quantitatively assessed but dichotomized as reperfusion or no-reperfusion by visual comparison of the admission and follow-up CTP images. Little is known about the quantitative assessment of reperfusion with CTP, and no universal thresholds for the assessment of change of CTP deficits are available. Moreover, the discrepancy rate for quantitative assessment of reperfusion with the TIMI reperfusion score on angiography has been described to be as high as 41 %, which seems to justify using a simple qualitative assessment [30]. Qualitative interpretation of CTP has shown good to excellent agreement rates between observers [31–33].

## Conclusion

The occurrence of hemorrhagic transformation does not seem to be associated with reperfusion. This suggests that other causes, like ischemic injury or the effects of IV-rtPA, are more important in the occurrence of hemorrhagic transformation in acute ischemic stroke.
